# Identification and expression profiles of sRNAs and their biogenesis and action-related genes in male and female cones of *Pinus tabuliformis*

**DOI:** 10.1186/s12864-015-1885-6

**Published:** 2015-09-15

**Authors:** Shi-Hui Niu, Chang Liu, Hu-Wei Yuan, Pei Li, Yue Li, Wei Li

**Affiliations:** National Engineering Laboratory for Forest Tree Breeding, College of Biological Science and Technology, Beijing Forestry University, Beijing, 100083 People’s Republic of China

**Keywords:** *Pinus tabuliformis* Carr, miRNA, siRNA, Parallel analysis of RNA ends (PARE), Male cones, Female cones

## Abstract

**Background:**

Small RNA (sRNA) play pivotal roles in reproductive development, and their biogenesis and action mechanisms are well characterised in angiosperm plants; however, corresponding studies in conifers are very limited. To improve our understanding of the roles of sRNA pathways in the reproductive development of conifers, the genes associated with sRNA biogenesis and action pathways were identified and analysed, and sRNA sequencing and parallel analysis of RNA ends (PARE) were performed in male and female cones of the Chinese pine (*Pinus tabuliformis*).

**Results:**

Based on high-quality reference transcriptomic sequences, 21 high-confidence homologues involved in sRNA biogenesis and action in *P. tabuliformis* were identified, including two different DCL3 genes and one AGO4 gene. More than 75 % of genes involved in sRNA biogenesis and action have higher expression levels in female than in male cones. Twenty-six microRNA (miRNA) families and 74 targets, including 46 24-nt sRNAs with a 5’ A, which are specifically expressed in male cones or female cones and probably bind to AGO4, were identified.

**Conclusions:**

The sRNA pathways have higher activity in female than in male cones, and the miRNA pathways are the main sRNA pathways in *P. tabuliformis.* The low level of 24-nt short-interfering RNAs in conifers is not caused by the absence of biogenesis-related genes or AGO-binding proteins, but most likely caused by the low accumulation of these key components. The identification of sRNAs and their targets, as well as genes associated with sRNA biogenesis and action, will provide a good starting point for investigations into the roles of sRNA pathways in cone development in conifers.

**Electronic supplementary material:**

The online version of this article (doi:10.1186/s12864-015-1885-6) contains supplementary material, which is available to authorized users.

## Background

The functional differentiation and adaptability to different environments of cells and tissues harbouring the same genetic material are dependent on epigenetic regulation at different levels. Small RNA (sRNA)-mediated gene silencing and chromatin modification play important roles in regulation [1]. The sRNA pathways in plants mainly include the microRNA (miRNA) and short-interfering RNA (siRNA) pathways [2]. According to the biogenesis and action mechanisms of sRNAs, the siRNA pathway is divided into trans-acting siRNA (tasiRNA), natural-antisense siRNA (natsiRNA) and RNA-directed DNA methylation (RdDM) pathways [3].

The miRNAs are a family of small endogenous noncoding single-stranded RNA molecules that regulate gene expression posttranscriptionally by directing mRNA degradation or translational repression and control many biological functions, including development and tissue-specific processes in both plants and animals [4, 5]. Plant miRNAs are generally 21 nucleotides long and regulate endogenous gene expression by recruiting silencing factors assembled into the RNA-induced silencing complex (RISC) to complementary binding sites in target transcripts [6, 7]. In most studied plants, such as Arabidopsis [8], rice [9], tomato [10], soybean [11], peanut [12], apple [13], miRNAs are the second most abundant sRNAs, followed by siRNAs [14]. siRNAs are distinguished from miRNAs in that they are derived from double-stranded RNA precursors. In plants, 24-nt siRNAs are associated with DNA methylation through the RdDM pathway at homologous loci guided by AGO4 proteins [15–18].

The sRNAs play a pivotal role in flower transformation and development [19, 20]. miR156 participates in ambient temperature-responsive flowering [21] and male fertility [22], miR159 controls anther development [23, 24] and pollen tube-synergid interaction [25], miR172 mediates sex determination and floral meristem determinacy [26–28], miR319 is required for petal development [29], and miR396 is involved in pistil development [30, 31]. Although there has been much work on the reproductive regulatory roles of miRNAs, there has been less emphasis on siRNAs. However, there is evidence that 24-nt siRNAs are probably critical in the regulation of flowering time [32], anthers [33], petals [34] and embryonic [35] development.

Despite this broad knowledge of sRNA biogenesis and the action mechanisms underlying growth and development of angiosperm plants, there is still a considerable lack of corresponding research on gymnosperms. With the popularisation of next-generation sequencing technology, sRNA sequencing and identification were also performed for some conifers [9, 36]. The sRNA expression profiles of infectious diseases [37], somatic embryonic induction and germination [38, 39], and male and female gametophytes [40, 41] were analysed in different conifer trees. However, these studies focused mainly on changes in expression of specific sRNAs, while research on the sRNA biogenesis and action pathways is very limited.

To improve our understanding of the roles of sRNA pathways in male and female cones of *Pinus tabuliformis*, the genes associated with sRNA biogenesis and action pathways were identified and analysed, and high-throughput sequencing of sRNAs and degradome tags of *P. tabuliformis* male and female cones was performed. These data provide compelling new insights into the regulation of sRNA pathways involved in male and female cone development in *P. tabuliformis*.

## Results

### Identification of homologues involved in sRNA biogenesis and action in *P. tabuliformis*

The sRNA biogenesis and action pathways are well defined in *Arabidopsis* [3]. Through a Blast search of the *P. tabuliformis* transcriptomic sequences [42] using the amino acid sequences of proteins from *Arabidopsis*, several highly similar sequences were selected and mapped to the *Picea abies* genome [43]. Specific screening primers were designed based on the longest sequence in each cluster to isolate the full-length sequences from the *P. tabuliformis* SMART cDNA library (Clonetech, USA). Finally, 24 candidate genes with complete coding regions were isolated, and the phylogenetic relationships between these *P. tabuliformis* genes and those of other land plants were inferred using the ML method. Surprisingly, the sRNA pathway genes were highly conserved during evolution, except for methyltransferases involved in the anRdDM pathway (Additional file 1). Twenty-one high-confidence homologues involved in sRNA biogenesis and action in *P. tabuliformis* were identified (Table 1).

**Table 1:** The sRNA pathway genes in *Pinus tabuliformis*

*At* gene	Locus	*Pt* homolog	NCBI NO.	Protein	Function
*AtHST*	At3g05040	*PtHST*	KJ711062	1195	Exprotin-5 homolog
*AtHEN1*	At4g29160	*PtHEN1*	KJ711060	977	sRNA-sprecific methyltransferase
*AtDRB4*	At3g62800	*PtDRB4*	KJ711042	550	nuclear dsRNA-binding protein
*AtHYL1*	At1g09700	*PtHYL1*	KJ711063	485	nuclear dsRNA-binding protein
*AtSGS3*	At5g23570	*PtSGS3*	KJ711106	776	Coiled-coil protein
*AtRDR1*	At1g14790	*PtRDR1*	KJ711100	1726	RNA-dependent RNA polymerase
*AtRDR2*	At4g11130	*PtRDR2*	KJ711101	1189	RNA-dependent RNA polymerase
*AtRDR6*	At3g49500	*PtRDR6*	KJ711102	1123	RNA-dependent RNA polymerase
*AtDCL1*	At1g01040	*PtDCL1*	KJ711036	2126	Rnase III
*AtDCL2*	At3g03300	*PtDCL2*	KJ711037	1435	Rnase III
*AtDCL3*	At3g43920	*PtDCL3a*	KJ711038	1871	Rnase III
*AtDCL3*	At3g43920	*PtDCL3b*	KJ711039	1792	Rnase III
*AtDCL4*	At5g20320	*PtDCL4*	KJ711040	1716	Rnase III
*AtNRPD1a*	At1g63020	*PtNRPD1a*	KJ711089	1856	DNA-dependent RNA polymerase
*AtNRPD1b*	At2g40030	*PtNRPD1b*	KJ711090	2530	DNA-dependent RNA polymerase
*AtNRPD2*	At3g23780	*PtNRPD2*	KJ711091	1348	DNA-dependent RNA polymerase
*AtAGO1*	At1g48410	*PtAGO1*	KJ710984	1144	RNA slice
*AtAGO2*	At1g31280	NA			RNA slice
*AtAGO3*	At1g31290	NA			RNA slice
*AtAGO4*	At2g27040	*PtAGO4*	KJ710986	930	RNA slice
*AtAGO5*	At2g27880	*PtAGO5*	KJ710987	1097	RNA slice
*AtAGO6*	At2g32940	*PtAGO4*	KJ710986	930	RNA slice
*AtAGO7*	At1g69440	*PtAGO7*	KJ710988	1127	RNA slice
*AtAGO9*	At5g21150	*PtAGO4*	KJ710986	930	RNA slice
*AtAGO10*	At5g43810	*PtAGO10*	KJ710985	955	RNA slice
*AtCMT3*	At1g69770	NA			Methyltransferase
*AtDRM2*	At5g15380	NA			Methyltransferase
*AtMET1*	A5t49160g	NA			Methyltransferase

### Two different *DCL3* genes exist in conifers

DCL enzymes are large proteins that catalyse primary sRNA transcript cleavage and produce mature sRNAs of different sizes [44]. Four different AtDCL enzymes were found in *Arabidopsis* and were divided into four groups, corresponding to DCLs from other plants. All four classes of DCLs exist in *P. tabuliformis*, indicating that they evolved before the divergence of angiosperms and gymnosperms (Additional file 1).

Different DCLs specifically process precursor transcripts into differently sized sRNAs. DCL1 and DCL4 generate 21-nt sRNAs, DCL2 generates 22-nt sRNAs, while DCL3 generates 24-nt sRNAs [45]. In angiosperms, the 24-nt sRNAs are the major endogenous sRNAs [9]; however, their levels are substantially lower in gymnosperms [43]. DCL3 was once considered to be absent in gymnosperm plants [46], but later studies suggest multiple DCL3 members exist in conifers [47].

**Fig. 1:**
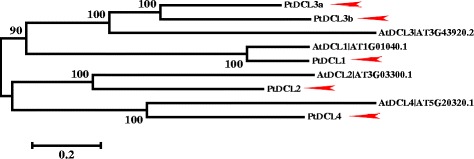
Phylogenetic analysis of PtDCL and AtDCL proteins. The figures show an unrooted maximum likelihood tree based on amino acid sequences. The gene names and IDs are provided to the right of each branch. The horizontal branch lengths are proportional to the estimated number of amino acid substitutions per residue. Bootstrap values were obtained from 1000 bootstrap replicates. The arrows indicate *P. tabuliformis* genes investigated in this study. The ML tree of DCL proteins from 42 land plants is shown in Additional file 1

Our results demonstrated two different *DCL3* genes in *P. tabuliformis* (Table 1, Fig. 1). The identities between the *PtDCL3a* and *PtDCL3b* cDNA sequences are only 68.5 %; however, the identity of *PtDCL3a* to its *Pinus taeda* and *Picea abies* homologues are 98 % and 94 %, respectively, while the identity of *PtDCL3b* to its homologues are 97.0 % and 93 %, respectively. These results indicate that *DCL3a* and *DCL3b* were separated for a long time before the divergence of conifer species.

### The AGO4s binding to the 24-nt DCL3-derived siRNAs were conserved during land plant evolution

**Fig. 2:**
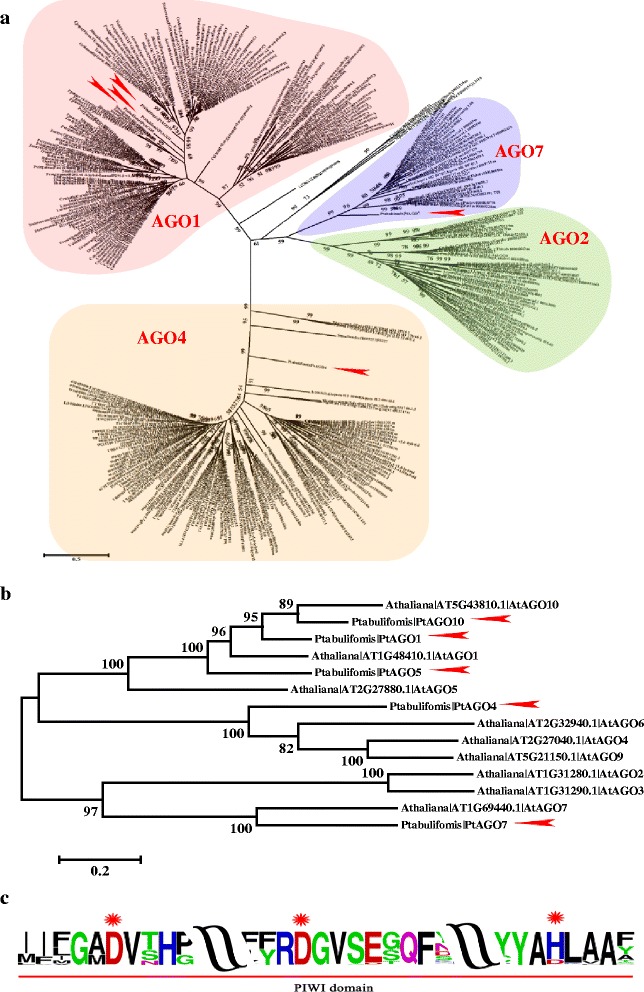
Phylogenetic analysis of AGO proteins in land plants. (**a**), The figures show an unrooted ML tree based on the amino acid sequences of all AGO proteins in land plants. (**b**), The figures show an unrooted ML tree based on the PtAGOs and AtAGOs. The horizontal branch lengths are proportional to the estimated number of amino acid substitutions per residue. Bootstrap values were obtained from 1000 bootstrap replicates. The arrows indicate the *P. tabuliformis* genes investigated in this study. (**c**) The catalytic DDH amino acid core in the PIWI domain of land plant AGOs. The sizes of letters represent the residue frequency of each site

AGO proteins are key components of the RNA-induced silencing complex (RISC) [48, 49]. Phytogenetic analyses showed that plant AGO proteins group into three clades (Fig. 2a). Five AGOs were found in *P. tabuliformis*. PtAGO1, 5, and 10 belong to the AGO1 clade, and PtAGO4 and PtAGO7 belong to the AGO4 and AGO7 clades, respectively (Fig. 2b). The catalytic DDH amino acid core in the PIWI domain of land plant AGOs was extremely conserved (Fig. 2c).

**Fig. 3:**
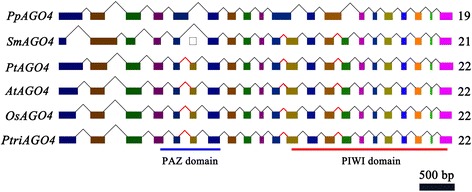
The gene structures and functional domains of land plant AGO4 genes. The AGO4 homologous structures in *P. patens*, *S. moellendorffii*, *P. tauliformis*, *A. thaliana*, *O. sativa* and *P. trichocarpa*. Coloured boxes represent different exons. The total numbers of exons are shown to the right side of the figure

Despite the fact that 24-nt DCL3-derived siRNAs are only present at very low levels in conifers [43] and that the AGO4 clade *ago* mutants in *Arabidopsis* (ago4, ago6, ago9) have no obvious developmental defects [48], AGO4s were conserved during land plant evolution. Moreover, the number, position, and size of exons of *AGO4* homologues in land plants remained surprisingly consistent (Fig. 3). Greater efforts are needed to understand the specific role of AGO4 in species maintenance and evolution.

### The sRNA biogenesis and action pathways have higher activity in female than in male cones of *P. tabuliformis*

**Fig. 4:**
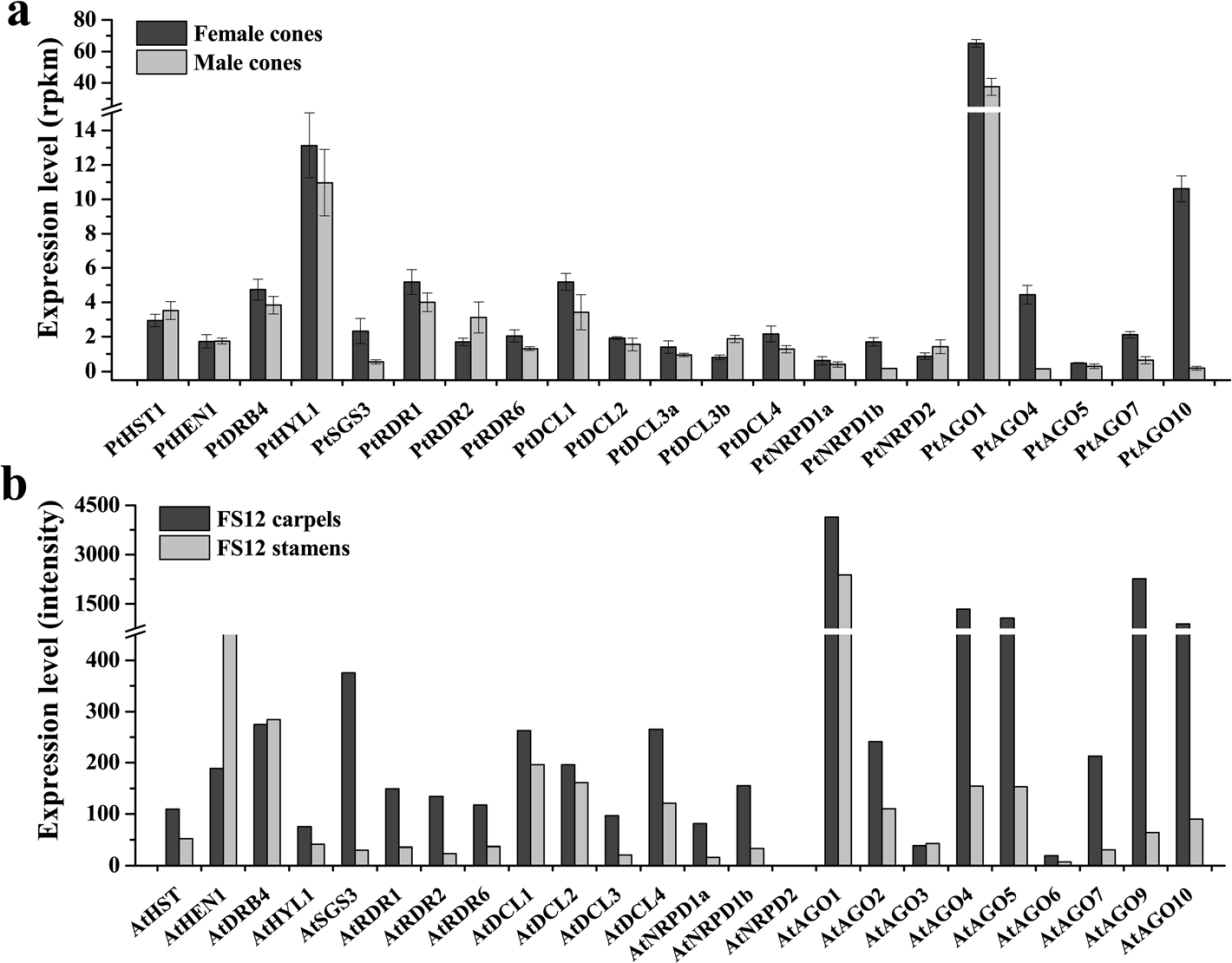
The expression patterns of genes involved in sRNA biogenesis and action pathways in male and female reproductive structures of *P. tabuliformis* and *A. thaliana*. (**a**), The expression patterns of *P. tabuliformis* genes in male and female cones. Bars indicate the means and standard errors of three biological replicates. (**b**), The expression patterns of *A. thaliana* genes in the stamens and carpels. FS12 indicates flower stage 12. The expression data were downloaded from the *A. thaliana* database (http://jsp.weigelworld.org/expviz/expviz.jsp)

The expression profiles of genes involved in the sRNA biogenesis and action pathways in male and female cones were analysed. The results show that more than 75 % of genes have higher expression levels in female than in male cones (Fig. 4a). These differences were confirmed by microarray data (Additional file 2). Interestingly, the female structures (carpels) in *Arabidopsis* also had similarly higher activities than those of the male structures (stamens) (Fig. 4b). Moreover, *AGO1* had the highest expression level, and *AGO4* and *AGO10* were highly differentially expressed between male and female structures in both *P. tabuliformis* and *Arabidopsis*, indicating that a similar sRNA regulatory mechanism probably underlies the development of male and female structures in both gymnosperms and angiosperms.

**Fig. 5:**
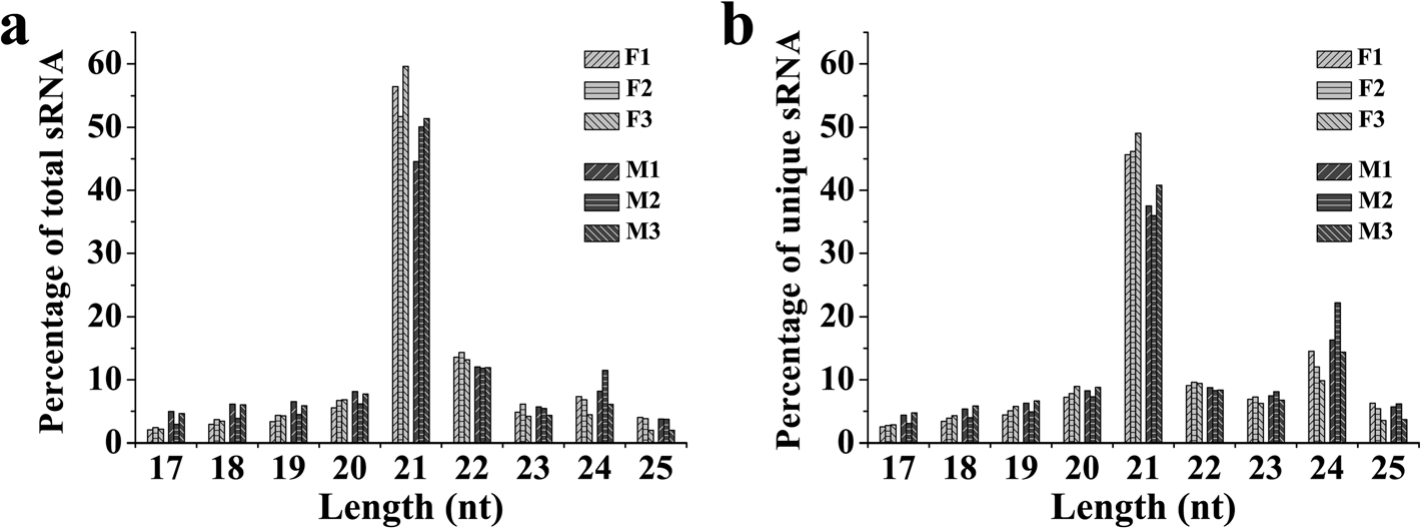
The sRNA length distribution in male and female cones of *P. tabuliformis*. (**a**) The length distribution of total sRNAs. (**b**) The length distribution of unique sRNAs. M1-3 and F1-3 indicate the three libraries of male cones and female cones, respectively

sRNAs in male and female cones were then analysed by high–throughput sequencing. The results showed that 21-nt sRNAs were the major sRNAs in both male and female cones in *P. tabuliformis*, with more in female than male cones (Fig. 5). Proportionally, the male cones had relatively high levels of 24-nt sRNAs (Fig. 5), but *AGO4,* which plays a key role in the action of 24-nt sRNAs, was expressed at a very low level in male cones (Fig. 4a), indicating that both miRNA and siRNA pathways have higher activities in female than male cones.

### Identification of miRNAs and targets in male and female cones of *P. tabuliformis*

To globally and directly identify miRNAs and miRNA-directed targets of cleavage, a parallel analysis of RNA ends (PARE), also known as degradome analysis, was applied. Twenty-six miRNA families and 74 targets were identified by sRNA sequencing and PARE analysis. Three novel miRNAs with unknown functions were isolated (Table 2, Additional file 3). When a two-fold change (FC) in expression was used to filter the differentially expressed miRNAs between male and female cones, 50 miRNAs were identified (Additional file 4). Eighteen genes had higher expression levels in male cones, while the other 32 miRNAs had higher expression levels in female cones (Additional file 4). This result is consistent with the sRNA biogenesis and action pathways having higher activities in female than in male cones in *P. tabuliformis* (Fig. 4).

**Table 2:** Experimental identified miRNA targets in *P. tabuliformis*

miRNA family	Target	Protein	Conserved target / miRNA sequence	Action sites
miR156	comp75271_c0_seq2	PtSPL1	[50]	Flowering
miR156	lw_isotig09062	PtSPL3	[50]	Flowering
miR529	comp85892_c0_seq1	PtSPL2	[76]	Flowering
miR159	lw_hbkxs4402jlyd6	PtMYB33	[51]	Flowering
miR162	comp74382_c0_seq3	PtDCL1	[52]	sRNA
miR172	comp64707_c0_seq1	PtAP2L3	[52]	Flowering
miR172	lw_isotig05156	PtAP2L2	[52]	Flowering
miR172	lw_isotig06154	PtAP2L1	[52]	Flowering
miR319	lw_isotig09509	PtTCP2	[53]	Flowering
miR319	lw_isotig09013	PtERF1	5’-TTGGACTGAAGGGAGCTCC-3’	
miR166	comp65619_c0_seq2	PtHB3*	[77]	Vascular
miR166	comp78056_c0_seq1	PtHB3*	[77]	Vascular
miR166	comp83755_c0_seq1	PtHB4	[77]	Vascular
miR166	lw_isotig05204	PtHB2	[77]	Vascular
miR169	comp77240_c0_seq3	PtNF-YA7	[78]	Root
miR171	comp65826_c0_seq1	PtHAM1	[79]	Meristem
miR171	lw_hbkxs4402gb5ou	PtHAM2	[79]	Meristem
miR391	comp48694_c0_seq1	unknown	5’-TACGCAGGAGAGATGACACCG-3’	
miR391	lw_isotig02711	unknown	5’-TACGCAGGAGAGATGACACCG-3’	
miR394	lw_isotig14380	PtKRF2	[80]	Stem cell
miR396	comp57471_c0_seq2	PtGRF2*	[31]	Flower/Pistil
miR396	comp73392_c0_seq1	PtGRF1	[31]	Flower/Pistil
miR396	lw_isotig04039	PtGRF3	[31]	Flower/Pistil
miR408	comp20033_c0_seq1	PtSINAT1	5’-TGCACTGCCTCTTCCCTGGCT-3’	
miR408	lw_isotig03980	PtAPRN	5’-TGCACTGCCTCTTCCCTGGCT-3’	
miR482	comp10992_c0_seq1	PtNBS1	[81]	Defense
miR482	comp2059_c0_seq1	unknown	5’-TCTTTCCTACTCCTCCCA-3’	
miR482	comp270247_c0_seq1	unknown	5’-TCTTCCCTACTCCTCCCATTCC-3’	
miR482	comp43645_c0_seq1	unknown	5’-TTTCCTACTCCTCCCAAGCCCA-3’	
miR482	comp57920_c0_seq1	unknown	5’-TTTCCTACTCCTCCCAAGCCCA-3’	
miR482	comp59077_c0_seq2	unknown	5’-TCTTGCCTACCCCTCCCATTCC-3’	
miR482	comp66603_c0_seq1	unknown	5’-TTTCCTACTCCTCCCAAGCCCA-3’	
miR482	comp76079_c0_seq1	unknown	5’-TCTTCCCTACTCCTCCCATTCC-3’	
miR482	comp80951_c0_seq5	unknown	5’-TTTCCTACTCCTCCCAAGCCCA-3’	
miR482	lw_isotig06642	PtKRF3	5’-TCTTCCCTACTCCTCCCATTCC-3’	
miR482	lw_isotig09777	unknown	5’-TCTTCCCTACTCCTCCCATTCC-3’	
miR482	lw_isotig12233	unknown	5’-TTCCCTATTCCTCCCATTCCTA-3’	
miR482	lw_isotig17369	unknown	5’-TCTTCCCTACTCCTCCCATTCC-3’	
miR482	lw_isotig25482	unknown	5’-TTTCCTACTCCTCCCAAGCCCA-3’	
miR946	comp74586_c0_seq1	un known	5’-CAGCCCTTCTCCTATCCACAAC-3’	
miR947	comp58863_c0_seq2	unknown	5’-CATCGGAATCTGTTACTGTTTC-3’	
miR947	comp69066_c0_seq2	unknown	5’-CATCGGAATCTGTTACTGTTTC-3’	
miR947	lw_hbkxs4402jaz6z	unknown	5’-CATCGGAATCTGTTACTGTTTC-3’	
miR947	lw_isotig08583	unknown	5’-CATCGGAATCTGTTACTGTTTC-3’	
miR949	comp29204_c0_seq1	unknown	5’-TCTCCGGGAATCCAATGCGCCT-3’	
miR949	comp4036_c0_seq1	unknown	5’-TCTCCGGGAATCCAATGCGCCT-3’	
miR950	comp314883_c0_seq1	NB-ARC	5’-TAACATCTGGGCCACGAGGGTT-3’	
miR950	lw_hbkxs4402g5r7f	unknown	5’-TCACATCTGGGCCACGATGGTT-3’	
miR951	comp77599_c0_seq2	unknown	5’-TGTTCTTGACGTCTGGACCACG-3’	
miR951	comp79416_c0_seq1	unknown	5’-TGTTCTTGACGTCTGGACCACG-3’	
miR951	comp79471_c2_seq6	unknown	5’-TCGGCCTCAAATGTTAGGAGAA-3’	
miR951	lw_hbkxs4401es9bl	unknown	5’-TGTTCTTGACGTCTGGACCACG-3’	
miR1311	lw_isotig09685	unknown	5’-TCAGAGTTTTGCCAGTTCCGCC-3’	
miR1312	comp141994_c0_seq1	PtGRF2*	5’-TTTGGAGAGAAAATGGCCACT-3’	
miR1312	comp78456_c0_seq1	PtHB1	5’-TTTGGAGAGAAAATGGCCACT-3’	
miR1313	comp70891_c0_seq2	PtLRK1	5’-TACCACTGAAATTATTGTTCG-3’	
miR1314	comp14858_c0_seq1	unknown	5’-CCGGCCTCAAATGTTAGGAGAA-3’	
miR1314	comp47488_c0_seq1	unknown	5’-CCGGCCTCAAATGTTAGGAGAA-3’	
miR1314	comp62379_c0_seq1	unknown	5’-CCGGCCTCAAATGTTAGGAGAA-3’	
miR1314	comp66316_c0_seq1	unknown	5’-CCGGCCTCAAATGTTAGGAGAA-3’	
miR1314	comp67690_c0_seq1	unknown	5’-CCGGCCTCGAATGTTAGGAGA-3’	
miR1314	comp77805_c0_seq6	unknown	5’-CCGGCCTCAAATGTTAGGAGAA-3’	
miR1314	comp78314_c0_seq1	PtRNAase	5’-CCGGCCTCAAATGTTAGGAGAA-3’	
miR1316	lw_isotig01063	PtLIP1*	5’-TTCCATGCACAAACCATTGGA-3’	
miR1316	lw_isotig22693	PtLIP1*	5’-TTCCATGCACAAACCATTGGA-3’	
miR1316	lw_isotig25086	PtLIP2	5’-TTCCATGCACAAACCATTGGA-3’	
miR1316	lw_isotig25889	PtLIP1*	5’-TTCCATGCACAAACCATTGGA-3’	
miR1448	lw_isotig17502	unknown	5’-TCTTTCCAACGCCTCCCATACC-3’	
miR2111	lw_isotig01996	PtKRF1	5’-TAATCTGCATCCTGAGGTTTG-3’	
miR2118	comp35426_c0_seq1	unknown	5’-TTCCCTATTCCACCCATCCCAT-3’	
miR3710	comp76797_c0_seq3	unknown	5’-TGAACAATGCCCACCCTTCATC-3’	
new	comp333751_c0_seq1	unknown	5’-TGACATTGTAAAATACGGGAAT-3’	
new	comp54693_c0_seq1	unknown	5’-TCAGGGCCTCGGTGGTTAATG-3’	
new	comp69194_c0_seq1	PtmTERF1	5’-TAATGCTTCACCCTCAATGCC-3’	

The miRNAs that shown in the table were isolation and sequencing from at least two independent libraries and the targets cleavage by miRNAs were identified by PARE analysis. * indicate the unigenes with same name were found as same gene after cloned

**Fig. 6:**
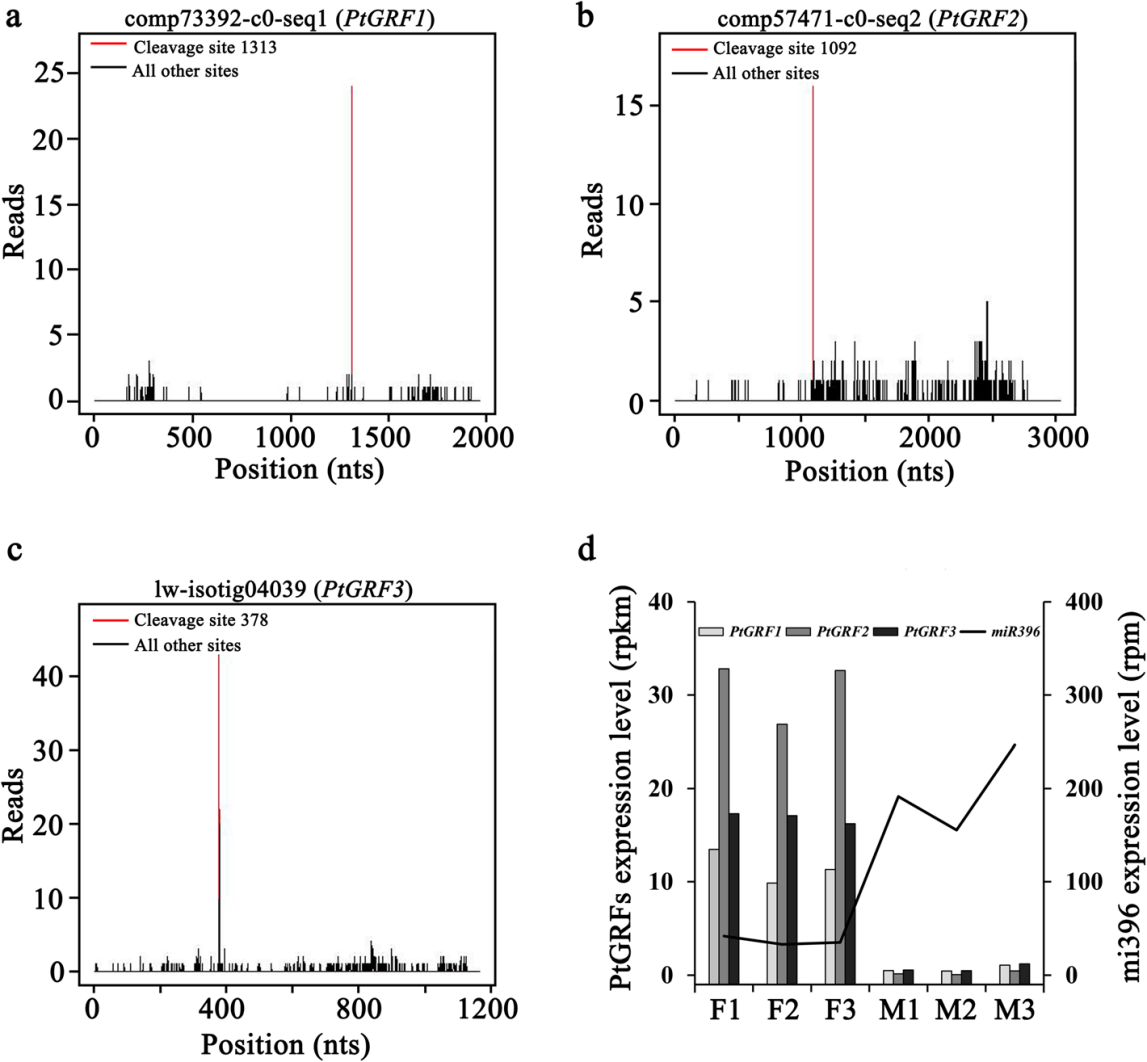
The cleavage and expression patterns of miR396 targets in male and female cones of *P. tabuliformis*. (**a**)-(**c**) Experimental identification of cleaved miR396 targets by miR396. (**d**) The expression patterns of miR396 and its targets in male and female cones of P. tabuliformis

The completed coding DNA sequences (CDSs) of 36 target genes were isolated, of which 20 miRNA targets were conserved in the evolution of conifers and angiosperms (Table 2). The important miRNA targets involved in angiosperm reproductive development, such as miR156/miR529-*SPLs* [50], miR159-*MYBs* [51], miR172-*AP2Ls* [52], miR319-*TCPs* [53] and miR396-*GRFs* [31], also exist in *P. tabuliformis* (Table 2, Additional file 5). The miR396-*GRFs* were previously found to be required for coordination of cell division and differentiation during leaf development [54, 55], and recent studies have shown that they also play a role in reproductive development [30, 31]. We isolated three *GRF* homologues from *P. tabuliformis*, namely *PtGRF1-3*, and miR396 mediated cleavage of the *PtGRFs* and regulated *PtGRF* mRNA accumulation (Fig. 6).

### Identification of 24-nt sRNAs containing a 5' “A” terminal differentially expressed between male and female cones in *P. tabuliformis*

Compared with the miRNA pathway, the role of the 24-nt siRNA-mediated RdDM pathway in the reproductive development of plants is largely unknown [48]. Only one AGO4 homologue, the key component of RISC associated with 24-nt siRNAs, was found in *P. tabuliformis* (Table 1, Fig. 2). Because AGO4 was revealed to predominantly bind 24-nt sRNAs with a 5’ A [56], the 24-nt sRNAs containing 5’ “A” termini differentially expressed between male and female cones of *P. tabuliformis* were identified. Eleven and 35 sRNAs specifically expressed in male and female cones, respectively, were isolated (Additional file 6). The functional identification of these 24-nt sRNAs in reproductive development will be instructive to our future research.

## Discussion

The sRNA-mediated transcriptional regulation of genes, including the miRNA and siRNA pathways, is an important epigenetic regulatory mechanism in plants [1]. In this study, we first isolated the key regulatory factors involved in miRNA and siRNA biogenesis and action in *P. tabuliformis*. Phylogenetic analysis indicated that sRNA pathways were very ancient regulatory mechanisms during the evolution of land plants, and most homologous genes, such as DCLs, AGOs and RDRs, had already diverged in the primitive vascular plants. However, the siRNA pathways probably evolved later than the miRNA pathways. The sRNA binding and guiding protein AGOs and the 24-nt siRNA-mediated DNA methylation catalytic genes have expanded and diversified in angiosperms [57].

In addition to the sRNA target genes, the sRNA biogenesis and action pathways also play important roles in the regulation of growth and development in plants [58, 59]. The expression profiles of the sRNA biogenesis and action pathway genes and sRNA sequencing indicated that the miRNA pathway is the main sRNA pathway in male and female cones of *P. tabuliformis*. Previous studies showed that the siRNA pathway has weak activity in other organs compared with cones [35, 43]. In angiosperms, the miRNA pathway is also the most important sRNA pathway in reproductive regulation [20]. Based on sRNA sequencing and PARE analysis, the cleavage of 74 target sequences by 26 corresponding miRNA families was identified. The complete CDS of 36 genes from these target sequences were cloned, while other genes were difficult to obtain by PCR as the mRNA of these genes was almost completely degraded by the high abundance of related miRNAs (average RPM > 3700) in the cones of *P. tabuliformis*. The roles of turn off of these genes in reproductive development remain unclear. It is noteworthy that we found that at least a portion of these genes were probably non-coding RNAs, and may be indirectly involved in developmental regulation.

Our results showed that the important miRNAs and their targets involved in angiosperm reproductive development, such as miR156/miR529-*SPLs* [50], miR159-*MYBs* [51], miR172-*AP2Ls* [52], miR319-*TCPs* [53] and miR396-*GRFs* [31], coevolved and have an ancient evolutionary history, similar to the sRNA pathways, such as miR156 and miRNA319, which have evolved in moss plants [60]. These miRNA-target-mediated regulatory pathways may have also coevolved as a "package", as *MYB33* is the target of miR159, which is predominantly expressed in the male reproductive structures in different species [23, 24].

DNA methylation is involved in the control of all genetic functions including transcription, replication, DNA repair, gene transposition and cell differentiation in plants [61]. It is a common and very ancient epigenetic regulatory mechanism in plants that is found in the DNA of all archegoniates investigated; however, the degree and features of DNA methylation are species-, tissue-, organelle- and age-specific [61]. 24-nt siRNA-mediated site-specific DNA methylation through the RdDM pathway is an important DNA methylation mechanism [62]. Previous studies suggested that gymnosperms have lower DNA methylation levels than those of flowering plants [63], which may be associated with the high degree of conservation and low morphological diversity between conifer species [43]. The 24-nt sRNAs involved in RdDM only represent a small proportion of all sRNAs in conifers [35, 43], but the proportions are opposite in the flowering plants [9]. Therefore, some researchers have speculated that the RdDM pathway in conifers is incomplete [46]. Our results have shown that, except for methyltransferase, all RdDM pathway components are present and conserved in *P. tabuliformis*, including PtDCL3, PtAGO4, PtRDR2, PtHEN1, PtNRPD1a, PtNRPD1b and PtNRPD2. The low level of 24-nt sRNAs is not because of a lack of biogenesis enzymes. The real reason may be, the low expression levels of RDR2-NRPD1a-DCL3 coding genes necessary for 24-nt sRNA accumulation.

AGO proteins are sRNA binding and guiding proteins and the most important proteins downstream of the sRNA pathways [64]. Despite the RdDM pathway having only weak activity in conifers, the components of RdDM were still conserved at a high degree through time. The structures of *AGO4* in moss, lycophyte, gymnosperm and angiosperm plants maintain a high level of consistency. Interestingly, the role of RdDM in mosses and lycophytes is unclear, as the *ago4* mutant has no obvious developmental defects [65, 66] and the evolutionary significance and selective pressure of the conservation of *AGO4* and RdDM is difficult to understand. Some evidence indicates that the absence of *AGO4* makes the plants more sensitive to disease [65]. Investigating the role of *PtAGO4* in *P. tabuliformis* in disease resistance may be valuable for understanding the role of RdDM in evolution and may facilitate disease resistant breeding of *P. tabuliformis.*

We found 46 24-nt sRNAs with a 5’ A that probably bind to AGO4 [56]. They were specifically expressed in either male cones or female cones, and more than 75 % of these sRNAs have significant accumulation in female cones but were not detected in all male samples. This is consistent with the higher activity of sRNA biogenesis and action pathway genes in female cones compared with male cones of *P. tabuliformis*. Because of the huge genome size, the analysis of large-scale genome methylation is difficult in conifers, and the function of these specifically expressed 24-nt sRNAs is unclear and deserves more attention in future studies.

## Conclusions

Based on high-quality reference transcriptome sequences [42], 21 high-confidence homologues involved in sRNA biogenesis and action in *P. tabuliformis* were identified. Phylogenetic analysis indicated that the sRNA pathways are highly conserved from mosses and ferns to higher plants. The expression profiles of these genes suggested that the sRNA pathways have higher activities in female than in male reproductive structures. In contrast to the angiosperms [14], both biogenesis- and action-related gene expression and sRNA sequencing revealed that the miRNAs are the most abundant sRNAs in *P. tabuliformis*, rather than siRNAs. In this study, 26 miRNA families and the miRNA-directed cleavage of 74 corresponding targets were identified though correlation analysis of sRNA and PARE sequencing data. The miRNAs and their targets participating in reproductive development in angiosperms, such as miR156-SPLs, miR159-MYBs, miR172- AP2Ls, miR319-TCP and miR396-GRFs, were also found in *P. tabuliformis*. They have ancient evolutionary histories similar to the sRNA pathways.

In conifers, the low level of 24-nt DCL3-derived siRNAs was not caused by the absence of DCL3 and AGO4. Two *DCL3* genes and one *AGO4* gene were found in *P. tabuliformis*, its ortholog PgAGO in *Picea glauca* [67] was previously identified. Forty six 24 nt sRNAs with a 5’ A, which probably bind to AGO4, specifically expressed in either male or female cones were isolated. The specific, highly expressed 24-nt sRNAs identified in conifers will provide a good starting point for investigations into the function and evolution of siRNAs in conifers.

## Methods

### Plant material and sample collection

*P. tabuliformis* immature male and female cones were collected from 3 individual trees selected at random (genetically distinct) in the botanic gardens in Beijing, China (116°33.9116′ E, 40°00.0861′ N and 44 m a.s.l.). Cones were sampled at 11:00 am on April 21, 2013. Each experiment was performed with at least three biological replicates per event. Samples were immediately placed in liquid nitrogen in the field after collection and all samples were stored at −80 °C in the laboratory before analysis.

### Identification of homologues involved in sRNA pathways in *P. tabuliformis*

Amino acid sequences of *Arabidopsis thaliana* genes (Table 1) were downloaded from the TAIR database (http://Arabidopsis.org). The protein sequences of *Arabidopsis* were used in queries to screen the *P. tabuliformis* transcriptome sequences (NCBI accession number SRA 056887) based on the TBLASTN method. The candidate sequences were selected and compared with other available conifer transcriptome sequences (http://dendrome.ucdavis.edu/resources/) and the *Picea abies* genome (http://congenie.org). The *P. tabuliformis* complete-length SMART cDNA library (Clonetech, USA) was screened using specific primers. The full-length sequences were obtained and compared with the original sequences. The nucleotide sequences of candidate genes were selected for preliminary phylogenetic analysis based on the NJ method using the MEGA software [68] and renamed.

### Phylogenetic analysis

Homologues of 41 land plant species, which have been whole genome sequenced (http://phytozome.jgi.doe.gov), were selected for phylogenetic analysis. Multiple alignments of protein sequences were obtained using the MUSCLE software [69] and a maximum-likelihood tree, based on the JTT model, was generated using MEGA software [68]. Bootstrap values were obtained from 1000 replicates.

### sRNA sequencing and PARE analysis

Total RNA isolation from samples and cDNA library construction were performed as described previously [39]. Pooled libraries were used for cluster generation on Illumina’s Cluster Station (Illumina, San Diego, USA) and then sequenced on an Illumina Hiseq2000 at YQYK-BIO (Beijing, China) following the vendor's recommended protocol. The sRNA abundance was measured as reads per million reads (RPM). The PARE library construction and sequencing were performed as described previously [70, 71]. The identification of miRNA and miRNA-directed targets of cleavage though correlation analysis of sRNA and PARE sequencing results was performed as previously described [72, 73]. More details are available in the supplementary material (Additional file 7).

### Gene expression analysis

RNA sequencing and gene expression analysis were described previously [74]. mRNA abundance was measured as reads per kilobase per million (RPKM) [75]. Each experiment was performed with at least three biological replicates per event. The mean RPKM of three biological replicates was compared among different samples.

### Identification of differentially expressed 24-nt sRNAs containing a 5’ "A" terminal between male and female cones

The 24-nt sRNAs containing a 5’ "A" terminal were extracted. Comparison of the expressions of these sRNAs was conducted between small RNA libraries of male and female cones. We first normalised the expression of sRNA in six libraries (F and M, three biological replicates each) to obtain the expression of reads per million reads (RPM). Then, the data were analysed using Fisher’s exact test with a Bonferroni correction for multiple hypothesis testing. Those sRNAs with a *p*-value below 0.01 and specifically expressed in either male cones or female cones were isolated.

## Additional files

Additional file 1:
**Phylogenetic relationships of land plant DCL-like, HEN1-like, HST-like, RDR-like and SGS3-like proteins.** (DOCX 267 kb) Additional file 2:
**The expression patterns of genes involved in sRNA biogenesis and action pathways in male and female reproductive structures of** ***P. tabuliformis*** **detected by microarray.** (DOCX 105 kb) Additional file 3:
**Experimental identification of cleaved miRNA targets.** (DOCX 2271 kb) Additional file 4:
**The differentially expressed miRNAs in male and female cones of** ***P. tabuliformis.*** (DOCX 27 kb) Additional file 5:
**Phylogenetic analysis of miR156/miR529, miR159, miR172, miR319 and miR396 regulated targets.** (DOCX 98 kb) Additional file 6:
**The differentially expressed 24-nt sRNAs containing a 5’ “A” terminal in male and female cones of** ***P. tabuliformis.*** (DOCX 20 kb) Additional file 7:
**Detailed description of methods.** (DOCX 25 kb)
