# Supplementary material for: Identification and expression profiles of sRNAs and their biogenesis and action-related genes in male and female cones of Pinus tabuliformis
Source: BMC Genomics. 2015 Sep 15;16(1):693. doi: 10.1186/s12864-015-1885-6 (PMC4570457; doi:10.1186/s12864-015-1885-6)

Figure S1 Phylogenetic relationships of land plants DCL-like proteins.


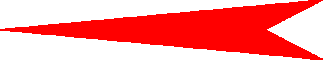

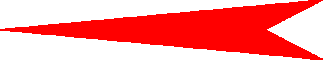

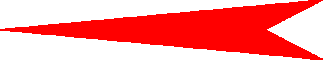

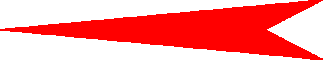

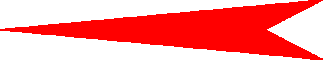


**DCL1**

**DCL2**

**DCL4**

**DCL3**

Figure S2 Phylogenetic relationships of land plant HEN1-like proteins.


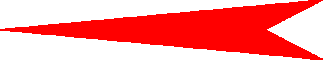


**Eduicots**

**Monocots**

**Gymnosperm**

Figure S3 Phylogenetic relationships of land plant HST-like proteins.

**Eduicots**

**Monocots**

**Gymnosperm**


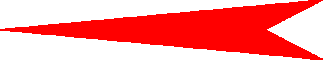


Figure S4 Phylogenetic relationships of land plant RDR-like proteins.


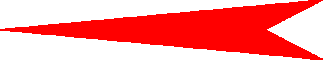

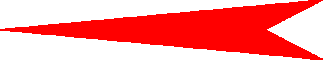

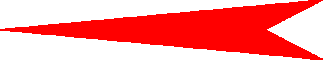


**RDR1**

**RDR2**

**RDR6**

Figure S5 Phylogenetic relationships of land plant SGS3-like proteins.

**Eduicots**

**Monocots**

**Gymnosperm**


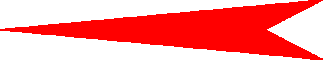

Supplement: Additional file 1: — Phylogenetic relationships of land plant DCL-like, HEN1-like, HST-like, RDR-like and SGS3-like proteins. (DOCX 267 kb) [file 12864_2015_1885_MOESM1_ESM.docx]
