# Supplementary figures and images for: Identification and expression profiles of sRNAs and their biogenesis and action-related genes in male and female cones of Pinus tabuliformis
Source: BMC Genomics. 2015 Sep 15;16(1):693. doi: 10.1186/s12864-015-1885-6 (PMC4570457; doi:10.1186/s12864-015-1885-6)

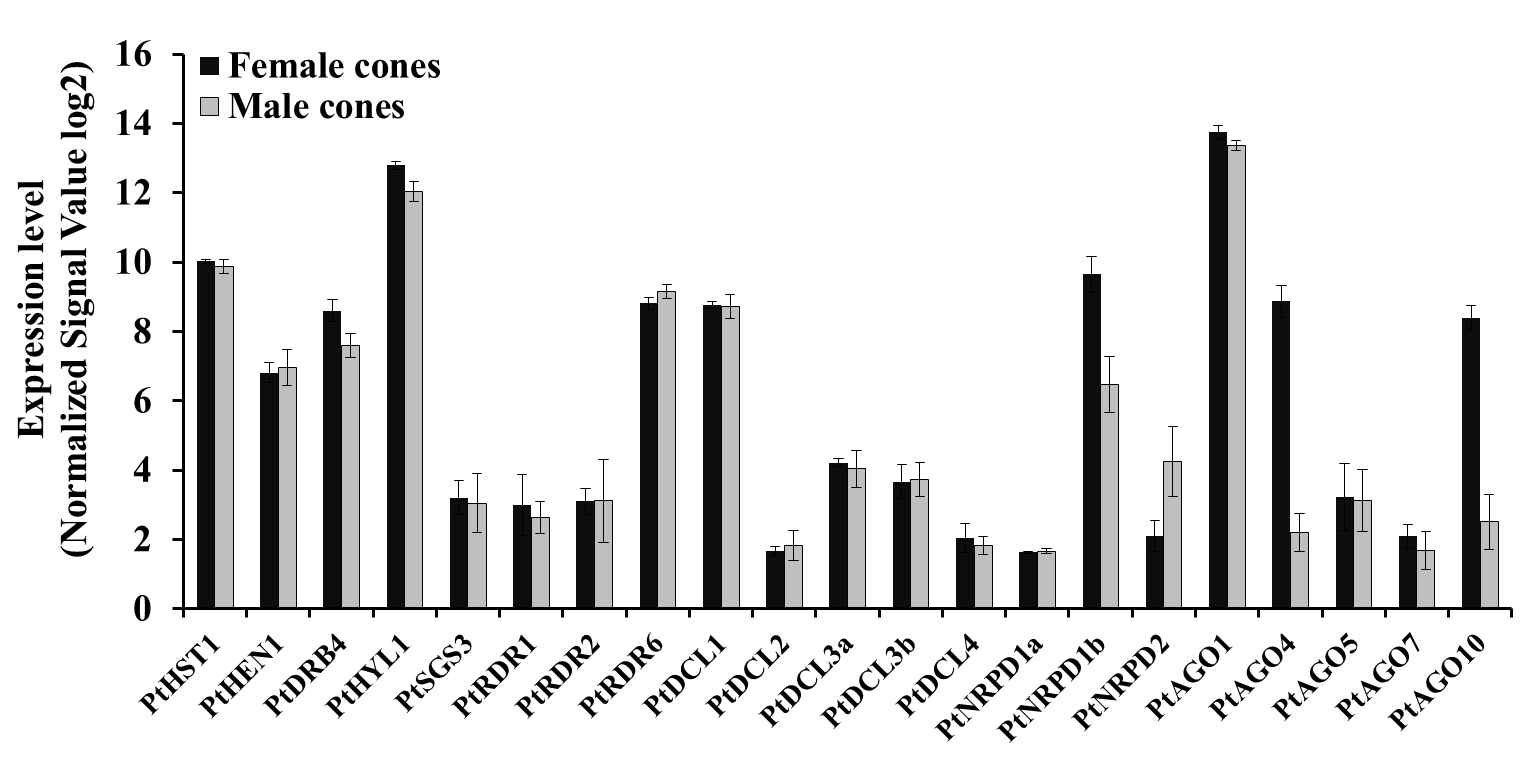

Supplement: Additional file 2: — The expression patterns of genes involved in sRNA biogenesis and action pathways in male and female reproductive structures of P. tabuliformis detected by microarray. (DOCX 105 kb) [file 12864_2015_1885_MOESM2_ESM.docx]

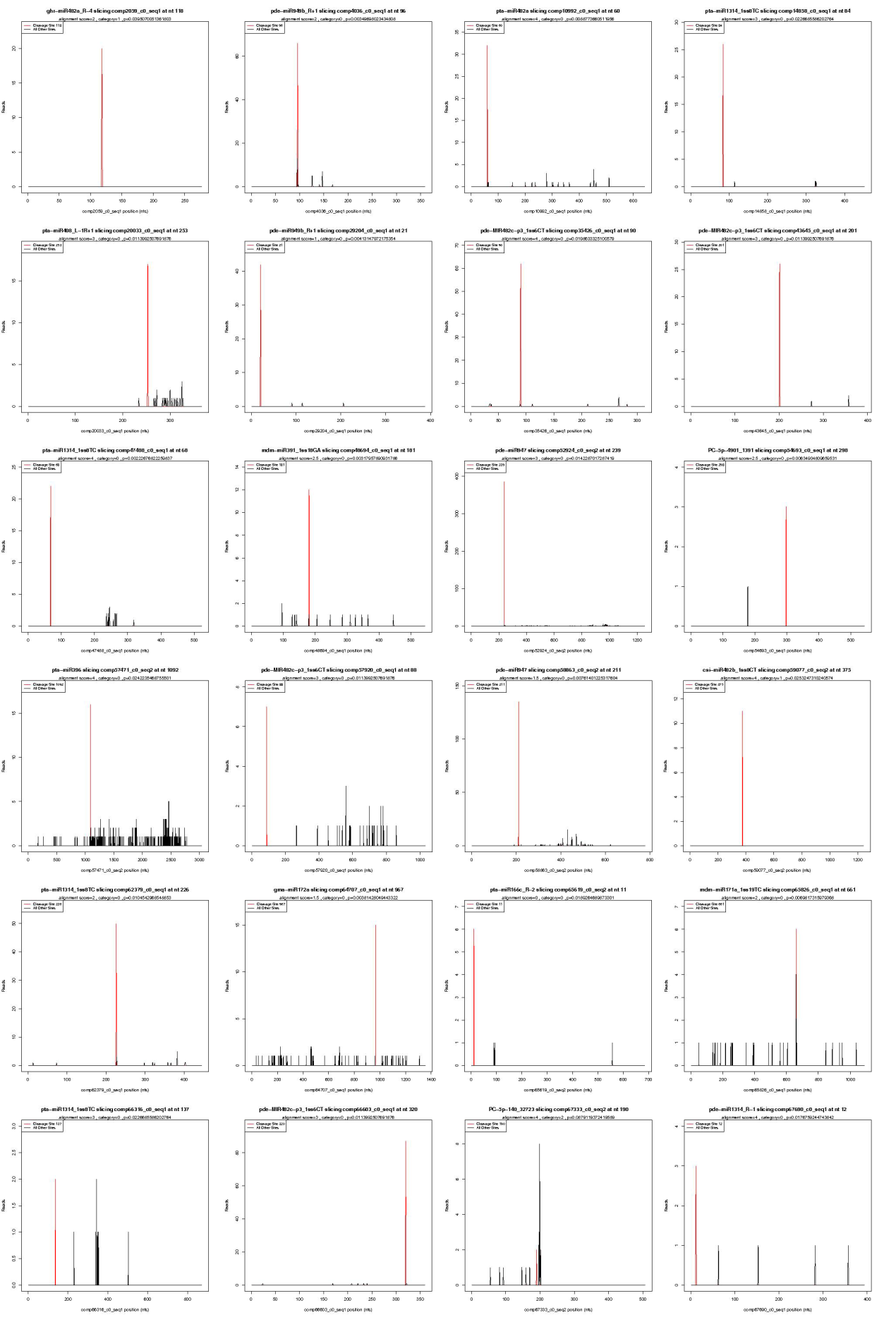

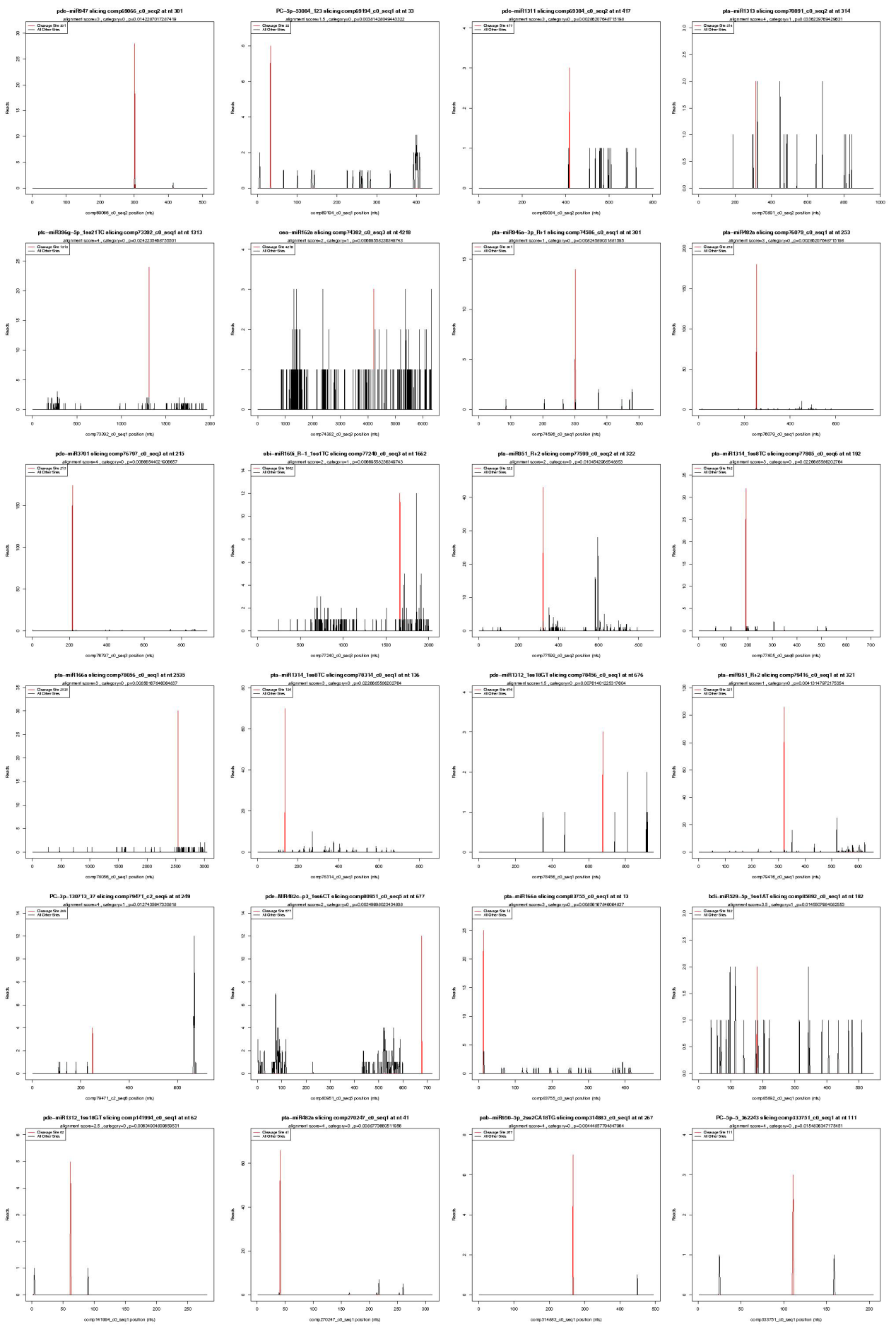

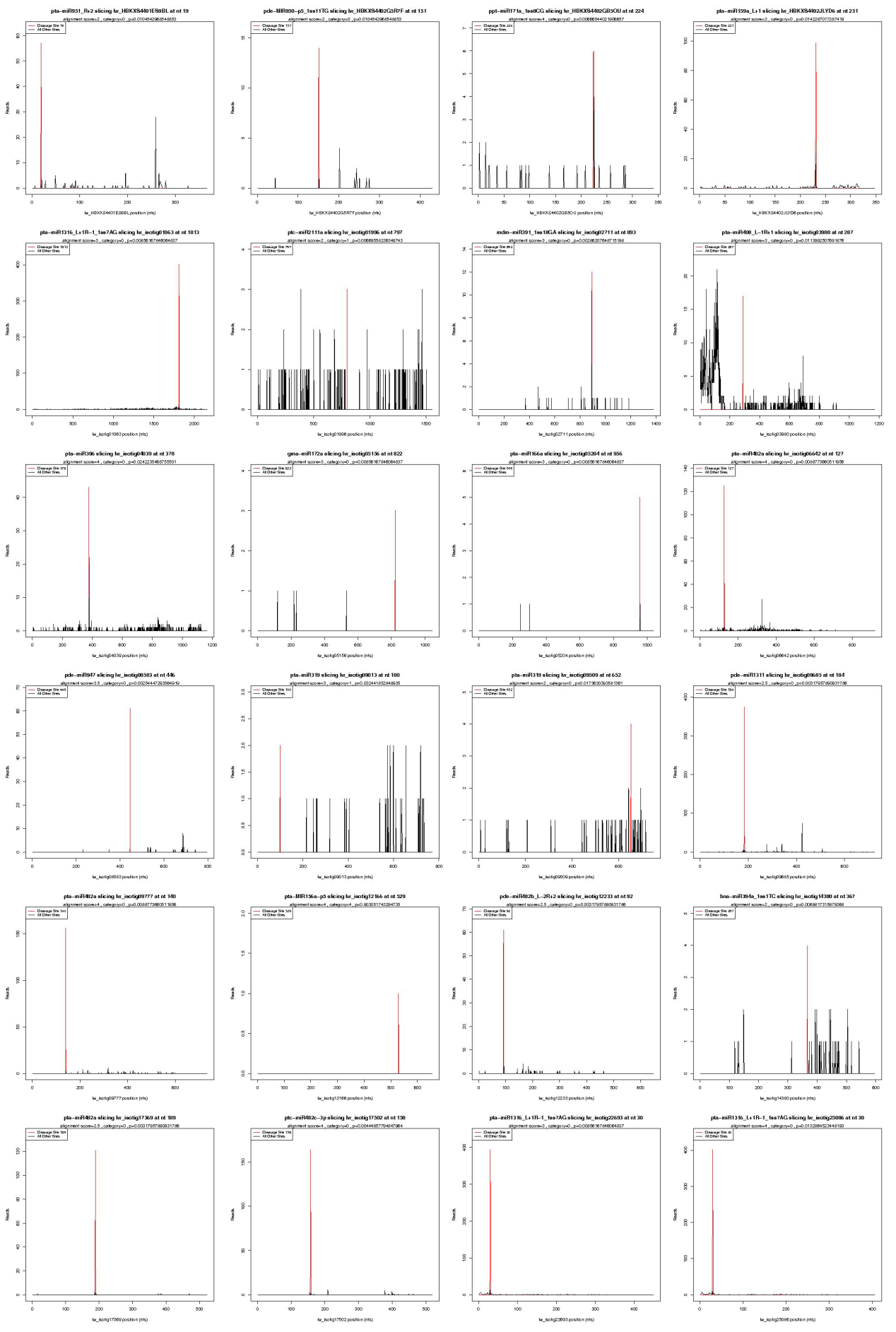

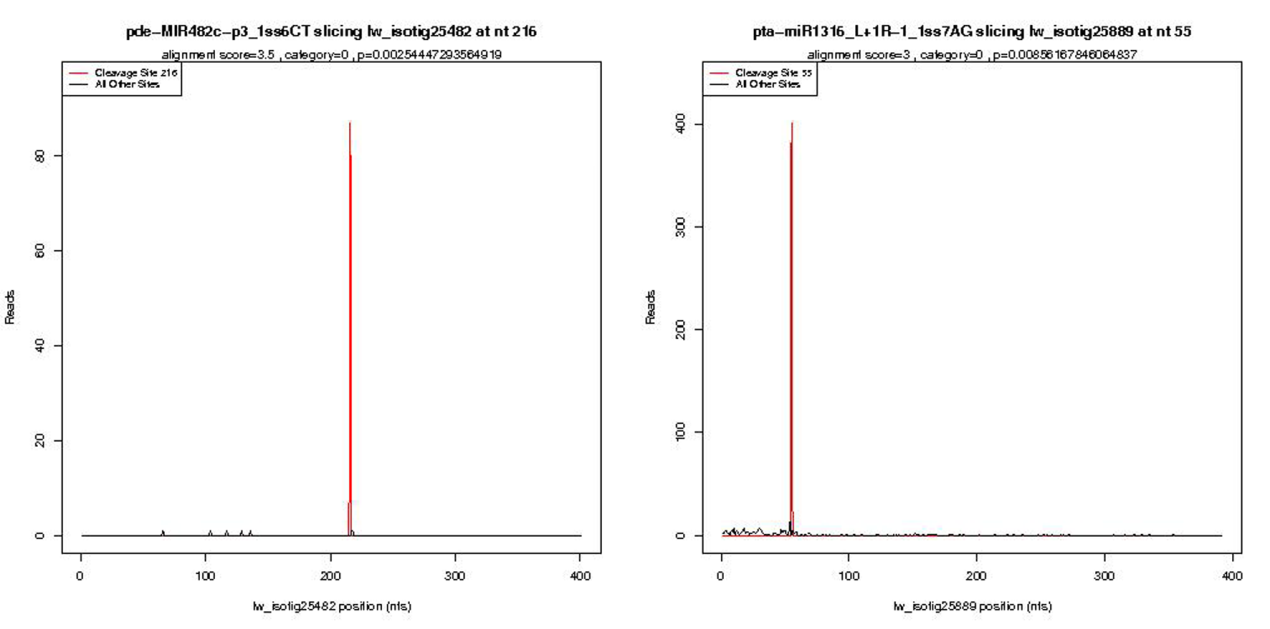

Supplement: Additional file 3: — Experimental identification of cleaved miRNA targets. (DOCX 2271 kb) [file 12864_2015_1885_MOESM3_ESM.docx]

**(a)**

**(b)**

**(c)**

**(d)**


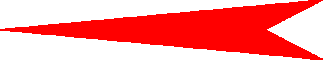

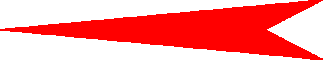

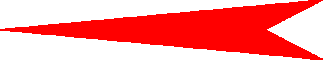

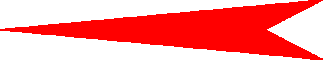

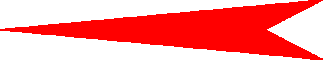

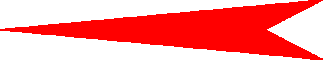

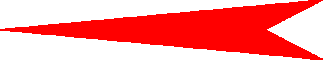

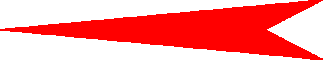


**(e)**


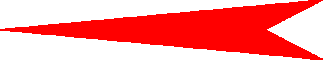

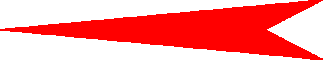

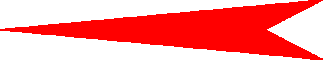

Supplement: Additional file 5: — Phylogenetic analysis of miR156/miR529, miR159, miR172, miR319 and miR396 regulated targets. (DOCX 98 kb) [file 12864_2015_1885_MOESM5_ESM.docx]
