# Supplementary material for: Identification and expression profiles of sRNAs and their biogenesis and action-related genes in male and female cones of Pinus tabuliformis
Source: BMC Genomics. 2015 Sep 15;16(1):693. doi: 10.1186/s12864-015-1885-6 (PMC4570457; doi:10.1186/s12864-015-1885-6)
Supplement: Additional file 4: — The differentially expressed miRNAs in male and female cones of P. tabuliformis. (DOCX 27 kb) [file 12864_2015_1885_MOESM4_ESM.docx]

The differentially expressed miRNAs in male and female cones of *P. tabuliformis*

| NO. | miRNA name | miRNA sequce | RPM(Female) | RPM(Male) | FC (log2) | *P* value |
| --- | --- | --- | --- | --- | --- | --- |
| 1 | pta-MIR159b-p5_1ss18CT | AACTGCTGGTTCATGGATTCT | 182±4.6 | 2±1.7 | -6.48 | 0.000 |
| 2 | ppt-miR529e_1ss1AT | TGAAGAGAGAGAGTACAGCCC | 0±0 | 88.1±11.5 | 6.46 | 0.008 |
| 3 | ptc-miR156a_L+1 | CTGACAGAAGAGAGTGAGCAC | 1.6±1.2 | 71.5±7 | 5.47 | 0.004 |
| 4 | ptc-miR159a_R-1 | TTTGGATTGAAGGGAGCTCT | 7859.5±1784.1 | 501.5±59.7 | -3.97 | 0.028 |
| 5 | ptc-miR390a_L+1 | TAAGCTCAGGAGGGATAGCGCC | 56±3.8 | 4±2 | -3.80 | 0.000 |
| 6 | ptc-miR390d-3p_1ss21AT | CGCTATCCATCCTGAGTTTTT | 10.9±1.9 | 0.6±0.2 | -3.44 | 0.017 |
| 7 | ptc-miR2111a | TAATCTGCATCCTGAGGTTTG | 158.4±29 | 16.7±9.7 | -3.24 | 0.013 |
| 8 | pta-miR1316_L+1R-1_1ss7AG | TTCCATGCACAAACCATTGGA | 15.3±2.9 | 141.3±1.3 | 3.21 | 0.000 |
| 9 | mtr-miR166g-5p | GGAATGTTGTCTGGCTCGAGG | 269.7±73 | 36.8±2.4 | -2.87 | 0.045 |
| 10 | mtr-miR319a-3p_R+4 | TTGGACTGAAGGGAGCTCCCTTTT | 9.3±0.9 | 1.4±1.2 | -2.78 | 0.002 |
| 11 | pta-MIR159a-p5 | TGGTTCAGCTGCTGATTCATG | 15.2±3.1 | 2.2±0.7 | -2.78 | 0.022 |
| 12 | pta-miR319 | TTGGACTGAAGGGAGCTCC | 14.1±2.1 | 2.2±0.8 | -2.70 | 0.008 |
| 13 | smo-miR1083_R+1_1ss20GT | TAGCCTGGAACGAAGCACGTTT | 6±1.1 | 0.6±0.4 | -2.58 | 0.013 |
| 14 | pab-miR396a | TTCCACAGCTTTCTTGAACTA | 36.3±3.8 | 197.7±37.5 | 2.44 | 0.025 |
| 15 | pab-miR535 | TGACAACGAGAGAGAGCACGC | 1103.5±374 | 5826±1155 | 2.40 | 0.021 |
| 16 | PC-3p-93737_57 | TGCACCTTGAGAGATTTGATC | 1.1±0 | 5.4±0.9 | 2.35 | 0.022 |
| 17 | PC-3p-2666_2479 | ATTCATTACAGTAGCCCAGTA | 155.6±12.2 | 783.3±92.3 | 2.33 | 0.010 |
| 18 | PC-5p-42070_161 | GTATCGATTGCGAGGCAAAGC | 18.2±0.7 | 3.7±1.6 | -2.31 | 0.002 |
| 19 | cln-miR166_R-2 | CCGGACCAGGCTTCATCCC | 4.9±1.3 | 0.9±0.5 | -2.30 | 0.033 |
| 20 | ppt-miR535a_R+1 | TGACAACGAGAGAGAGCACGCT | 32±4.2 | 152.9±5.2 | 2.26 | 0.000 |
| 21 | mdm-miR171a_1ss19TC | TTGAGCCGCGTCAATATCCCC | 4.7±1.1 | 1±0.9 | -2.20 | 0.022 |
| 22 | mtr-miR399l_2ss1TC5AG | CGCCGAAGGAGAGTTGCCCTG | 135±20.7 | 30.2±17.7 | -2.16 | 0.006 |
| 23 | pde-miR949b_R+1 | TCTCCGGGAATCCAATGCGCCT | 7197.5±879.5 | 1622.6±446.1 | -2.15 | 0.004 |
| 24 | rco-miR535_R+3 | TGACAACGAGAGAGAGCACGCTTT | 7.6±5.1 | 33±6.2 | 2.12 | 0.012 |
| 25 | PC-3p-639644_5 | AACCAGTTGTTCTGACCCAACATC | 0.2±0.3 | 4.3±0.3 | 2.11 | 0.000 |
| 26 | pde-MIR949b-p3 | TTAGGAGGAAGGTGTATTGAT | 607.2±77.5 | 148.9±48.8 | -2.03 | 0.004 |
| 27 | PC-5p-32681_221 | TGCAGACTGCCTGGCACGATT | 17.6±1 | 4.7±2.8 | -1.91 | 0.014 |
| 28 | pta-MIR159b-p3_1ss14AG | ACTTGGATTGAAGGGAGCTCC | 3.7±0.3 | 0.8±0.6 | -1.90 | 0.006 |
| 29 | bdi-miR529-5p_1ss1AT | TGAAGAGAGAGAGTACAGCCT | 0.6±0.6 | 3.7±0.6 | 1.90 | 0.007 |
| 30 | ppt-miR536f_L-1R+2 | TCGTGCCAAGCTGTGTGCATC | 13±0.9 | 3.5±0.8 | -1.90 | 0.000 |
| 31 | nta-miR172d | AGAATCTTGATGATGCTGCAT | 14.4±3 | 4.1±0.2 | -1.80 | 0.039 |
| 32 | pde-miR390 | AAGCCCAGGATGGATAGCGCC | 3.4±0.3 | 0±0 | -1.77 | 0.003 |
| 33 | PC-3p-563381_6 | CATGTCAGGCAGCATTTACTT | 0.8±0.6 | 3±0.7 | 1.60 | 0.029 |
| 34 | pta-miR159c_R-1 | CTTGGATTGAAGGGAGCTCC | 674±134.5 | 228.8±118.6 | -1.56 | 0.025 |
| 35 | pde-miR1314_R-1 | CCGGCCTCGAATGTTAGGAGA | 57.7±4.1 | 20±4.4 | -1.53 | 0.001 |
| 36 | PC-5p-3308_1974 | TCCCGGGAATCCAATGGGCCTT | 309.2±27.4 | 111.7±52.7 | -1.47 | 0.018 |
| 37 | mtr-miR396a-3p_L-1 | CTCAAGAAAGCTGTGGGAGA | 119.8±10.6 | 43.8±2.2 | -1.45 | 0.007 |
| 38 | pde-miR1314_R+1 | CCGGCCTCGAATGTTAGGAGAAT | 78.5±6.1 | 29.6±9.5 | -1.41 | 0.006 |
| 39 | rco-miR167c | TGAAGCTGCCAGCATGATCTGG | 1978.4±191.7 | 750.4±48.7 | -1.40 | 0.009 |
| 40 | pta-miR159a_L+1 | CTTGGATTGAAGGGAGCTCCA | 2042.1±349.8 | 805.4±434.8 | -1.34 | 0.037 |
| 41 | PC-5p-140_32723 | CTGGGCGACCGTAATGAATCC | 1107.1±250.5 | 2698.3±389.9 | 1.29 | 0.012 |
| 42 | PC-3p-30181_244 | TTGCACCTTGAGAGATTTGAT | 20±7.1 | 45.6±5.7 | 1.19 | 0.019 |
| 43 | PC-5p-53084_123 | TAATGCTTCACCCTCAATGCC | 5.8±2.4 | 13.2±2.4 | 1.19 | 0.037 |
| 44 | pde-miR1314 | CCGGCCTCGAATGTTAGGAGAA | 2751.3±207.9 | 1209.4±410.8 | -1.19 | 0.019 |
| 45 | PC-5p-7335_979 | TAATGCTTCACCCTCAATGCCC | 32.4±3.1 | 72.6±7.3 | 1.16 | 0.007 |
| 46 | ptc-miR393a-5p_R+1 | TCCAAAGGGATCGCATTGATCT | 0.2±0.3 | 2.2±0.4 | 1.14 | 0.009 |
| 47 | mdm-miR408a_L-1R+3 | TGCACTGCCTCTTCCCTGGCTTT | 2.2±0.4 | 0.4±0.3 | -1.12 | 0.011 |
| 48 | PC-3p-1459908_2 | GCGCCATCATGGAACCAAAAAC | 0.3±0.5 | 2.1±0.2 | 1.09 | 0.022 |
| 49 | pde-miR1314_R+1_1ss1CT | TCGGCCTCGAATGTTAGGAGAAT | 437.1±14.9 | 209.2±63.9 | -1.06 | 0.032 |
| 50 | pab-MIR396a-p3_1ss20AT | CTCAAGAAAGCTGTGGGAAT | 56±3.9 | 27.5±0.9 | -1.02 | 0.007 |
