# Supplementary material for: Identification and expression profiles of sRNAs and their biogenesis and action-related genes in male and female cones of Pinus tabuliformis
Source: BMC Genomics. 2015 Sep 15;16(1):693. doi: 10.1186/s12864-015-1885-6 (PMC4570457; doi:10.1186/s12864-015-1885-6)
Supplement: Additional file 6: — The differentially expressed 24-nt sRNAs containing a 5’ “A” terminal in male and female cones of P. tabuliformis. (DOCX 20 kb) [file 12864_2015_1885_MOESM6_ESM.docx]

The differentially expressed 24nt sRNAs containing “A” 5’ terminal in male and female cones of *P. tabuliformis*

| NO. | 24nt sRNA | RPM (Female) | | RPM (male) | |
| --- | --- | --- | --- | --- | --- |
| 1 | ACGAGACCAATTGCTCAGCTGGAT | | 441.8±87.5 | 0.0 |  |
| 2 | AAGATTACTACAACCTTGAGCGCT | | 260.4±27.7 | 0.0 |  |
| 3 | ATGAGTTTGAATATGTTGGAGGAT | | 176.1±73.7 | 0.0 |  |
| 4 | ATTAGAATTGAGGAAATGGTCACC | | 166.3±41.8 | 0.0 |  |
| 5 | AAACGCTCAAGGTTGTAGTAATCT | | 162.8±57.8 | 0.0 |  |
| 6 | AAAAGGACAAATAGATCGCTGGCT | | 161±54.3 | 0.0 |  |
| 7 | AGTTAGATGGCAAAATGTATACAA | | 158.8±25.7 | 0.0 |  |
| 8 | ACGTATTTCGTTTGCCGGCATCCC | | 149.5±32.5 | 0.0 |  |
| 9 | ACACGTATTTCGTTTGCCGGCATC | | 149±36.8 | 0.0 |  |
| 10 | AAATTCTGTAGCACAGATCACCCT | | 147.2±39.2 | 0.0 |  |
| 11 | AAAAAGATGACGAGGATTTGAAAA | | 136.4±79.1 | 0.0 |  |
| 12 | ACTCAAGAACGTGCTCAACGAGTT | | 133.7±72.2 | 0.0 |  |
| 13 | ACTCCCTGAAGATTTCGATCGCCT | | 115.1±47.9 | 0.0 |  |
| 14 | AGGACAAATAGATCGCTGGCTGGC | | 113.2±22.4 | 0.0 |  |
| 15 | AACTTCGCTCCCAGATCTGTAGAT | | 109.6±27.7 | 0.0 |  |
| 16 | ACGGTGATTGATAAAAACCTGGTA | | 109.4±39 | 0.0 |  |
| 17 | AAAAATTTAAAAAGATGACGAGGA | | 109.4±46.1 | 0.0 |  |
| 18 | AGTTGCAGAAGGAGACTGCGGGAC | | 105.1±13.9 | 0.0 |  |
| 19 | ATGCAAGTTGGATCGCCCTGACCA | | 104.8±18.4 | 0.0 |  |
| 20 | ATTGCGATGGTCCCTGCGGATGCT | | 103±27.5 | 0.0 |  |
| 21 | ATTTGCAAACCGCGACCGTGAAGC | | 100.8±15.5 | 0.0 |  |
| 22 | ATATTTATCGCAGAGCTGGGCAGC | | 98.1±30.8 | 0.0 |  |
| 23 | AACGCGGTCCATTCCATATACACC | | 97±29.9 | 0.0 |  |
| 24 | ATAAAAAAGGACAAATAGATCGCT | | 95.6±40.8 | 0.0 |  |
| 25 | ATTGGTGATAGTTGGTTAAGGACA | | 95.2±16.9 | 0.0 |  |
| 26 | AAGGGTGCACTCTGCACGAGCATC | | 94.5±31 | 0.0 |  |
| 27 | AAAGATCGCACAATAAACCAAACC | | 91.6±27.4 | 0.0 |  |
| 28 | AAAATCGGGAATTCACTAAGCACT | | 89.2±23.9 | 0.0 |  |
| 29 | ACAGATTTAACATGGCAATAGAGA | | 87.8±17.6 | 0.0 |  |
| 30 | AAGCTGTAGCGTTGAGATCTTTGA | | 86.8±21.6 | 0.0 |  |
| 31 | AAACCGCGACCGTGAAGCATCTCG | | 85.6±20.3 | 0.0 |  |
| 32 | ACTAGATTTTAGATCACCAGAGAT | | 85.4±19.7 | 0.0 |  |
| 33 | AACGAGACCAATTGCTCAGCTGGA | | 84.5±4.7 | 0.0 |  |
| 34 | ATAGTAATTCAACTTAGTACGAGA | | 82.6±15.5 | 0.0 |  |
| 35 | ACACAGATGAATCGGTGAGACGAA | | 77.8±10.3 | 0.0 |  |
| 36 | ACGGACCAAAACCCATATTGCTTT | | 0.0 | 82.8±8.2 |  |
| 37 | ATCCTAACAGACCGAGAGATTTGA | | 0.0 | 78.6±16.4 |  |
| 38 | ATATGGGCTCTGATGAAGGTGACT | | 0.0 | 101.8±25.5 |  |
| 39 | AGACAAAATGAAGAAAGATGAAAC | | 0.0 | 114.8±8 |  |
| 40 | ATAAACATGATCAGAAACTGAGCT | | 0.0 | 123.2±40.3 |  |
| 41 | AACCAAGGATGAGTAAGAGGCTCT | | 0.0 | 126.5±49.3 |  |
| 42 | ATTTCTGAAATATGTAAAGACTCT | | 0.0 | 163.7±69 |  |
| 43 | AGTTTTGTGAAGACTGTATAGGGT | | 0.0 | 191.8±64.3 |  |
| 44 | ACTTGGGATAAATGGGACATTACT | | 0.0 | 230.7±92.7 |  |
| 45 | AAGAAGTGGATACAAGACTGGGGA | | 0.0 | 235.6±47.1 |  |
| 46 | AGGAATGTTACAAGAGTTGGAGGA | | 0.0 | 266.6±76.3 |  |
