# Supplementary material for: Identification and expression profiles of sRNAs and their biogenesis and action-related genes in male and female cones of Pinus tabuliformis
Source: BMC Genomics. 2015 Sep 15;16(1):693. doi: 10.1186/s12864-015-1885-6 (PMC4570457; doi:10.1186/s12864-015-1885-6)
Supplement: Additional file 7: — Detailed description of methods. (DOCX 25 kb) [file 12864_2015_1885_MOESM7_ESM.docx]

**Construction of small RNA libraries and high throughput sequencing**

Small RNA libraries for male (M) and female (F) cones were generate from total RNAs using the Illumina Truseq small RNA preparation kit (RS-930-1012, Illumina Inc., USA) according to the preparation guide. The purified cDNA libraries were used for cluster generation on Illumina’s cluster station (Illumina, San Diego, USA) and then sequenced on an Illumina Hiseq2000 following the vendor's recommended protocol. The high throughput sequencing was performed at Beijing Yuanquanyike Biological Technology Co.,Ltd. (Beijing, China)

**Bioinformatic analyses of miRNAs**

The sequencing data were processed with a proprietary pipeline script, ACGT101-miR v4.2 (Beijing Yuanquanyike Biological Technology Co.,Ltd., Beijing, China). A series of digital filters (Beijing Yuanquanyike Biological Technology Co.,Ltd., Beijing, China) were first employed to remove various un-mappable sequencing reads. Then, the remaining sequences with lengths between 17 and 25bp were grouped by families (unique seqs) and were used to map with reference database files. Various mappings were performed on unique seqs against pre-miRNA and mature miRNA sequences listed in the miRBase 21.0 (June 26, 2014). Methods and criteria used for various mappings were documented in the ACGT-101 User’s Manual. The unique seqs were aligned to mRNA, RFam, and Repbase to remove mRNA, rRNA, tRNA, snRNA, snoRNA, repeat sequences, and other ncRNAs. In addition, unique seqs were predicted as novel miRNAs if they met the following criteria: (1) unique seqs were not mapped to the pre-miRNAs of selected species in the miRBase; (2) but the unique seqs were mapped to the genome; (3) and the extended sequences at the mapped genome positions had the propensity to form hairpins.

**Identification of different expressed miRNAs between male and female cones**

Comparison of miRNAs expression was made between small RNA libraries of male cones and female cones libraries. We first normalized the expression of miRNA in the six libraries (F and M, three biological replicates) to get the expression of reads per million reads (RPM). RPM= actual miRNA count / total count of clean reads× 10^6^. Then, the data were analyzed through Fisher’s exact test using a Bonferonni correction for multiple hypothesis testing. Those miRNAs having a *p*-value below 0.05 and a fold change greater than 2 were considered differentially expressed.

**PARE library preparation and sequencing**

Total RNA of male and female cones were extracted using Trizol reagent (Invitrogen, CA, USA) following the manufacturer’s procedure. The total RNA quantity and purity were analysis of Bioanalyzer 2100 and RNA 6000 Nano LabChip Kit (Agilent, CA, USA) with RIN number >7.0. Approximately 20 ug of total RNA were used to prepare PARE library. The method followed as [1, 2] with some modification. (1) Approximately 150 ng of poly(A)+ RNA was used as input RNA and annealing with Biotinylated Random Primers. (2) Strapavidin capture of RNA fragments through Biotinylated Random Primers. (3) 5’ adaptor ligation to only those RNAs containing 5’-monophosphates. (4) Reverse transcription and PCR (5) Libraries were sequenced using the 5’ adapter only, resulting in the sequencing of the first 36 nucleotides of the inserts that represented the 5’ ends of the original RNAs. And then we performed the single-end sequencing (36 bp) on an Illumina Hiseq2500 at the Beijing Yuanquanyike Biological Technology Co.,Ltd. (Beijing, China) following the vendor’s recommended protocol.

Raw sequencing reads were obtained using Illumina’s Pipeline v1.5 software following sequencing image analysis by Pipeline Firecrest Module and base-calling by Pipeline Bustard Module. A Public software package, CleaveLand 3.0 was used for analyzing sequencing data generated. The key functions performed by this software and the relevant analysis results are described here. (1) Get some degradome data (RNAseq reads from 5’ ends of uncapped RNAs, where the 1st nt of the read represents the in vivo 5’ end), free from adapter sequences, etc. (2) Map the degradome reads to the appropriate transcriptome using appropriate thresholds. (3) Filter the mapped data. (4) Summarize the mapped degradome data into a ”degradome density file” using script ’CleaveLand3 map2dd.pl’. (5) Generate small RNA / mRNA alignments using ’targetfinder.pl’. (6) Compare the degradome density file to the target predictions, and output significant hits using script ’CleaveLand3 analysis.pl’. (7) Generate ”t–plots” of the targets using script ’CleaveLand3 t– plotter.pl’.

**Gene expression analysis**

Total RNA was extracted using the Trizol reagent for gene expression analysis (Invitrogen, CA, USA) following the manufacturer’s procedure. Total RNA quantity and purity were assessed using the Bioanalyzer 2100 (Agilent, CA, USA). The mRNA was isolated with poly-T oligo attached magnetic beads from approximately 10 μg of total RNA (Invitrogen, USA). Following purification, the mRNA was fragmented into small pieces using divalent cations at an elevated temperature. Cleaved RNA fragments were reverse-transcribed to create the final cDNA library in accordance with the protocol for the mRNA-Seq sample preparation kit; the average insert size for paired-end libraries was 200-300 bp (Illumina, San Diego, USA). Pooled libraries were sequenced on the Illumina Hiseq2000 platform (Illumina, San Diego, USA). mRNA abundance was measured as reads per kilobase per million reads (RPKM), as described by [3]. Each experiment was performed with at least three biological replicates per event. The mean RPKM of three biological replicates were compared between different samples.

**References:**

1. Addo-Quaye C, Eshoo TW, Bartel DP, Axtell MJ: **Endogenous siRNA and miRNA targets identified by sequencing of the Arabidopsis degradome**. *CURR BIOL* 2008, **18**(10):758-762.

2. German MA, Luo S, Schroth G, Meyers BC, Green PJ: **Construction of Parallel Analysis of RNA Ends (PARE) libraries for the study of cleaved miRNA targets and the RNA degradome**. *NAT PROTOC* 2009, **4**(3):356-362.

3. Mortazavi A, Williams BA, McCue K, Schaeffer L, Wold B: **Mapping and quantifying mammalian transcriptomes by RNA-Seq**. *NAT METHODS* 2008, **5**(7):621-628.

**校对报告**

当前使用的样式是 [BMC Genomics]

当前文档包含的题录共3条

有0条题录存在必填字段内容缺失的问题

所有题录的数据正常
